# *Bacteroides Fragilis* Polysaccharide A Ameliorates Abnormal Voriconazole Metabolism Accompanied With the Inhibition of TLR4/NF-κB Pathway

**DOI:** 10.3389/fphar.2021.663325

**Published:** 2021-04-15

**Authors:** Xiaokang Wang, Chunxiao Ye, Tianrong Xun, Liqian Mo, Yong Tong, Wensi Ni, Suping Huang, Bin Liu, Xia Zhan, Xixiao Yang

**Affiliations:** ^1^ Department of Pharmacy, Shenzhen Hospital, Southern Medical University, Shenzhen, China; ^2^ School of Traditional Chinese Medicine, Southern Medical University, Guangzhou, China; ^3^ Department of Pharmacy, Shenzhen Longhua District Central Hospital, Shenzhen, China; ^4^ Department of Pharmacy, Nanfang Hospital, Southern Medical University, Guangzhou, China; ^5^ Department of Hematology, Shenzhen Hospital, Southern Medical University, Shenzhen, China; ^6^ Department of Pediatric, Shenzhen University General Hospital, Shenzhen, China; ^7^ Department of Intensive Care Unit, Shenzhen Longhua District Central Hospital, Shenzhen, China

**Keywords:** voriconazole, abnormal metabolism, polysaccharide A, TLR4 (toll-like receptor 4), hepatic inflammation

## Abstract

The antifungal agent voriconazole (VRC) exhibits extreme inter-individual and intra-individual variation in terms of its clinical efficacy and toxicity. Inflammation, as reflected by C-reactive protein (CRP) concentrations, significantly affects the metabolic ratio and trough concentrations of voriconazole. *Bacteroides fragilis* (*B. fragilis*) is an important component of the human intestinal microbiota. Clinical data have shown that *B. fragilis* abundance is comparatively higher in patients not presenting with adverse drug reactions, and inflammatory cytokine (IL-1β) levels are negatively correlated with *B. fragilis* abundance. *B. fragilis* natural product capsular polysaccharide A (PSA) prevents various inflammatory disorders. We tested the hypothesis that PSA ameliorates abnormal voriconazole metabolism by inhibiting inflammation. Germ-free animals were administered PSA intragastrically for 5 days after lipopolysaccharide (LPS) stimulation. Their blood and liver tissues were collected to measure VRC concentrations. PSA administration dramatically improved the resolution phase of LPS-induced hepatic VRC metabolism and inflammatory factor secretion. It reversed inflammatory lesions and alleviated hepatic pro-inflammatory factor secretion. Both *in vitro* and *in vivo* data demonstrate that PSA reversed LPS-induced IL-1β secretion, downregulated the TLR4/NF-κB signaling pathway and upregulated CYP2C19 and P-gp. To the best of our knowledge, this study is the first to show that PSA from the probiotic *B. fragilis* ameliorates abnormal voriconazole metabolism by inhibiting TLR4-mediated NF-κB transcription and regulating drug metabolizing enzyme and transporter expression. Thus, PSA could serve as a clinical adjunct therapy.

## Introduction

The liver maintains homeostasis and plays important roles in host defense and drug metabolism. Gut microbiota modulate the injury response in certain organs that are remote from the gastrointestinal tract, such as the liver (Gong et al., 2019). Intestinal blood accounts for 70% of the total hepatic blood supply. Gut microbiota composition affects hepatic cellular function (Adolph et al., 2018). Alteration of gut microbiota composition or target bacteria modifies the toxicity profile of certain drugs (Sivan et al., 2015; Taylor et al., 2019). Elucidating the physiological, chemical and microbial factors that determine the contributions of the microbiome to drug metabolism could help explain intra- and inter-individual variability in drug exposure and provide opportunities for personalized medicine (Zimmermann et al., 2019).

The fungal infection rates in immunocompromised patients with acute myeloid leukemia (AML) may be in the range of 8–12% (Hicks et al., 2020). Prophylactic administration of the antifungal agent VRC, reduces the incidence of invasive fungal infections. The pharmacokinetics of VRC are nonlinear and highly variable. Changes in trough concentrations (C_min_) are associated with genetic factors, drug interactions and pathophysiological parameters, such as age or hepatic function. Pharmacogenetics is a key component of precision medicine (Hicks et al., 2020), however, alterations in disease status can result in inter- and intra-individual drug exposure variability and many adverse drug reactions (ADRs) such as hepatotoxicity, loss of visual acuity, and neuropathies (Pascual et al., 2012). Inflammation is reflected by CRP concentrations and affects drug metabolic enzymes and transporters (DMETs) (Stanke-Labesque et al., 2020). It may also contribute to the pharmacokinetic variability of VRC. During infection or inflammation, several drug-metabolizing enzymes in the liver are downregulated (Morgan, 2009; Keller et al., 2016; Veringa et al., 2017). Consequently, VRC overexposure can occur in type two diabetes, non-alcoholic fatty–liver disease and hematopathies (Stanke-Labesque et al., 2020). However, there have been limited studies of VRC pharmacology based on patients’ inflammatory state and few have formulated or tested anti-inflammatory agents to improve hepatic DMETs.

Pro-inflammatory cytokines such as IL-6, IL-1β and TNF-α evoke major recombination in hepatic gene expression and induce the synthesis of acute phase proteins such as CRP. Toll-like receptors (TLRs) recognize patterns and are expressed in monocytes, macrophages, hepatocytes, and other cells (Kawai and Akira, 2010). As adjuvant, TLRs agonists can launch a strong immune response to assist cancer radiotherapy and bio-chemotherapy.

*B. fragilis* is a strict anaerobe in the human gut microbiome. Its natural product capsular PSA prevents certain sterile PNS and CNS inflammatory disorders (Mazmanian et al., 2008; Ramakrishna et al., 2019). PSA is a unique zwitterionic polysaccharide and an immunogenic capsule component of the non-toxin-producing *B. fragilis* (NTBF) strain NCTC9343. It is a unique T-cell-dependent antigen that mediates CD4^+^ T-cell proliferation and induces secretion of the immunosuppressive cytokine IL-10 in regulatory T cells. IL-10 is a potent anti-inflammatory cytokine that restrains pathogenic inflammation in the gut, brain and lungs (Lee and Mazmanian, 2010; Shen et al., 2012). A recent biodiversity study showed a clear relationship between NTBF and the immune system and this association implies its potential use as a probiotic (Sun et al., 2019). Therefore, we infer that NTBF may inhibit the inflammatory environment in the liver and the entire body by promoting interactions between microorganisms and hosts and ameliorating abnormal VRC metabolism in inflammatory disease.

## Materials and Methods

### Study Population

The study procedures were approved by the Ethics Committee of Shenzhen Hospital of Southern Medical University (No. NYSZYYEC20180017), and were performed in accordance with the Helsinki Declaration. Subjects were selected from patients seen in the Hematology Department of Shenzhen Hospital, Southern Medical University who were diagnosed with AML and required VRC for antifungal prophylaxis. AML was diagnosed according to the criteria of the National Comprehensive Cancer Network (NCCN) Guidelines for Acute Myeloid Leukemia (Tallman et al., 2019). All adult patients were tested for the CYP2C19 genotype before being grouped. Patients who were being administered “3 + 7” chemotherapy (3 days daunorubicin plus 7 days continuous cytarabine infusion) were screened for enrollment. Sixty-five patients with the CYP2C19 genotype who were normal metabolizers signed informed-consent forms before participating in this study.

The course of VRC treatment lasted ≥ 5 days. Each patient was administered two doses a day in strict accordance with drug-label instructions. The exclusion criteria were as follows: patients 1) who were pregnant within the past year or who were administered antibiotics or probiotics within the past 2 months; 2) who were <16 year old; 3) for whom combination chemotherapy was contraindicated because of renal/hepatic or cardiac insufficiency; 4) administered combinations of patent CYP2C19 or CYP3A4 modulators such as rifampicin, ritonavir, carbamazepine, long-acting barbiturates, or macrolide antibiotics; 5) who presented with hypokalaemia (serum potassium < 3.5 mmol/L) or those undergoing haemodialysis or peritoneal dialysis; 6) who presented with visual impairment; 7) who presented with hepatopathy; and 8) who presented with poor compliance to their medications according to electronic medical records.

### Clinical Data and Patient Blood Sample Collection

Clinical information was collected and ADRs were recorded in an earlier study (Wang et al., 2020). A 5 ml sodium -heparin tube was used for blood sample collection. CRP and TNF-α concentrations were used as indices of inflammation and were routinely measured in discarded blood samples. Serum total bilirubin (TB > 26 μmol/L), aspartate transaminase (AST > 40 IU/L), and alanine transaminase (ALT > 50 IU/L) were quantitated to diagnose hepatotoxicity during VRC treatment. The metabolic ratio was determined as follows:

$$\text{Metabolic ratio}=\frac{\text{C }\left(\text{Voriconazole N}-\text{oxide}\right)}{\text{C }\left(\text{Voriconazole}\right)}\times 100\text{ \%}$$

### Liquid Chromatography-Tandem Mass Spectrometry Analysis

Lipid LC-MS/MS was conducted as previously described (Wang et al., 2020). A Waters UPLC-TOF-MS (Waters ACQUITY UPLC™ System Tandem Waters LCT Premier XE TOF-MS; Waters Corp., Milford, MA, United States) was usedto determine plasma VRC and VRC N-oxide.

### Microbial Diversity Analysis

Human stool samples were collected from patients in the haematology department of Shenzhen Hospital, Southern Medical University. Faeces were collected in labelled stool tubes, flash-frozen, and stored at −80°C.

Microbial DNA was extracted from all fecal samples using an E. Z.N.A.® DNA kit (Omega Bio-Tek, Norcross, GA, United States) according to the manufacturer’s instructions. The V3 - V4 hypervariable region of the bacterial 16S rRNA gene was amplified by thermal cycling PCR with 338F_806R primers. All primers are listed in Supplementary Table S1. The purified amplification products were mixed in equimolar ratios and subjected to paired-end sequencing (2 × 300) on the Illumina MiSeq platform (Illumina, San Diego, CA, United States) according to standard methods. The original FASTQ files were demultiplexed, quality filtered with Trimmomatic, and flash merged. The RDP classifier algorithm (http://rdp.cme.msu.edu/) was used to classify each 16S rRNA gene sequence in the SILVA (SSU123) 16S rRNA database.

To investigate the roles of gut microbiota in adverse reaction development and modulation in patients, we analysed metagenomic sequence data extracted from the NCBI Sequencing Read Archive database using faecal samples from individuals with and without adverse reactions. DNA extraction and quality control were performed as previously described (Taur et al., 2012). Covaris M220 (Covaris Inc., Woburn, MA, United States) was used to cut, the extracted DNA fragments into 400-bp sections. The latter were then used to construct a double-terminal library. NEXTFLEX Rapid DNA-Seq (PerkinElmer, Waltham, MA, United States) was used to build a pair of terminal libraries. The joint containing the hybridisation site of the complete sequencing primer was connected to the blunt end of the fragment. A NovaSeq kit (Illumina, San Diego, CA, United States) was used on the Illumina NovaSeq platform to perform paired-end sequencing according to the manufacturer’s instructions (www.illlighta.com).

## Materials

LPS and TAK-242 were purchased from MCE (Monmouth Junction, NJ, United States). Enzyme-linked immunosorbent assay (ELISA) kits were purchased from Group, Inc (Rosemont, IL, United States). Primary antibodies against P-gp were obtained from Cell Signaling Technology (Danvers, MA, United States). His-H3 was procured from Affinity Biosciences (Cincinnati, OH, United States). Other antibodies were acquired from Invitrogen (Carlsbad, CA, United States).

### Animal Model and Treatments

Male Sprague-Dawley rats (180–220 g) and male C57BL/10J mice were purchased from Southern Medical University. They were housed in ventilated boxes, in a protected temperature- and humidity-controlled room, with a 12 h on/off light cycle. The animals had *ad libitum* food and water to access. Animal care was conducted in strict accordance with the China and Nanfang Hospital policies for animal health and well-being and the animals were euthanized by CO_2_ asphyxiation at the end of the experiments (No. NFYY-2020–73).

Germ-free mice were prepared by administering vancomycin (100 mg/kg), neomycin sulphate (200 mg/kg), metronidazole (200 mg/kg), and ampicillin (200 mg/kg) intragastrically once daily for 5 days.

The rats and mice were randomly assigned to control (CON), lipopolysaccharide (LPS), LPS + germ free, LPS + germ free + PSA (*n* = 6, respectively) groups. In the CON group, physiological saline (2 ml/kg) was continuously infused *via* the jugular vein. In the LPS group, LPS in saline was continuously infused *via* the jugular artery at a dose of 2 mg/kg bodyweight per hour. In the LPS + PSA group, PSA was diluted with sterile phosphate-buffered saline (PBS; vehicle) and infused *via* the jugular vein (2 ml/kg), starting 10 min before the LPS infusion. All of the germ-free animals were kept under sterile conditions, and received sterile nutrition and water. After 3 days of treatment, the animals were humanely euthanized by CO_2_ asphyxiation.

### Voriconazole Metabolism *In Vivo*

Blood and liver tissues were collected in heparinized test tubes at 0, 0.5, 2 and 12 h after VRC administration. Plasma was separated by centrifugation at 13,000 rpm for 10 min. VRC concentrations were determined by lipid LC-MS/MS.

### Polysaccharide A Purification

*B. fragilis* strain NCTC9343 was obtained from the American Type Culture Collection (Manassas, VA, United States). PSA purification was performed by Fenghai Biotechnology Co., Ltd. (Hangzhou, China) according to the method described previously (Ramakrishna et al., 2019). Bacteria were resuspended in 1:1 v/v water: phenol and centrifuged into fragments at 70°C. The aqueous phase was collected, extracted with ether, and dialyzed against water overnight. PSA was then digested twice with DNA and RNA enzymes and analyzed using 1H NMR (Supplementary Figure S2). Fractions containing PSA alone were collected, freeze-dried and stored at −80°C until use.

### Histologic Analysis

Isolated liver tissue was fixed in 10% (v/v) buffered formalin, embedded in paraffin, and sliced into 5 mm thick sections. The degree of liver impairment was assessed by H&E staining. The hepatic parameters were inflammation, coagulation, and lipid accumulation. For the immunohistochemical (IHC) analysis, monoclonal IL-1β antibody (1:1,000) was used.

### Primary Hepatocytes and Treatments

In brief, liver samples were immediately moved to a 10 cm sterile Petri dish and minced, then hepatocytes were collected using a large-bore pipette, filtered through a 70 μm membrane to remove tissue fragments, and transferred to sterile 10 cm cell-culture dishes. Primary hepatocytes were cultured in cold Dulbecco’s Modified Eagle’s Medium (DMEM), and seeded in 6 cm dishes containing DMEM supplemented with 10% fetal bovine serum. Cells were maintained at 37°C and 5% CO_2_ in a humidified atmosphere. Primary hepatocytes were incubated with LPS (0.5 µ g/mL) for 12 h to obtain inflammation. For PSA treatment, the cells were treated with 10 μg/ml for 2 h before LPS stimulation.

For the experiments studying the effects of inhibitor on TLR4/NF-κB induced IL-1β secretion, the cells were prestimulated with TAK-242 (1 μM) and added to the cell culture for 30 min at 2 h before stimulation with LPS.

Primary hepatocytes were seeded in 24-well plates. TLR4 specific siRNA (siTLR4) and its negative control siRNA (siNC) were purchased from RiboBio Co., Ltd. (Guangzhou, China). More effective single siRNAs were used for further experiments as follows: siTLR4#1: CTA​CTA​CCT​CGA​TGA​TAT​T; siTLR4#2: GGA​AAC​TTG​GAA​AAG​TTT​G. Non-specific negative control siRNAs were also designed (sense strand: 5′-UUC​UCC​GAA​CGU​GUC​ACG-3′ and anti-sense strand: 5′-ACG​UGA​CAC​GUU​CGG​AGA​ATT-3′). Lipofectamine 3,000 transfection reagent (Invitrogen, United States) was used for co-transfection with 40 nM siTLR4#2 and siNC according to the manufacturer's recommendations. After 48 h, the cells were collected and subjected to subsequent experimentation.

### Gene Expression Analysis

TRIzol reagent was used for total RNA isolation, and reverse transcription was performed using reverse transcriptase. Hepatic TLR2, TLR4, and TLR9 expression was quantified by RT-PCR.

### Cytokine Detection by Enzyme-Linked Immunosorbent Assay and Luminex Analysis

Serum and supernatant levels of IL-1β and IFN-γ were analyzed using ELISA kits (Nanjing Jiancheng Bioengineering Institute, Nanjing, China) according to the instructions of the manufacturer. IL-2, IL-6, IL-10, TNF-α and CXCL8 in serum were quantified by Luminex xMAP technology on an LX200 instrument (Luminex Corp., Austin, TX).

### Western Blotting Analysis

Protein extracts from whole cell and liver tissue samples were separated on SDS-PAGE gels and transferred onto polyvinylidene fluoride (PVDF) membranes (Millipore, Massachusetts, MA, United States). Total hepatic protein extraction was conducted using the radioimmunoprecipitation assay (RIPA) method. The membrane was sealed with 5% (w/v) skim milk in TBST buffer for 1 h and incubated at 4°C for 12 h with primary antibody diluted with 5% (w/v) skim milk TBST followed by secondary antibody coupled with horseradish peroxidase incubated with 5% (w/v) skim milk-TBST for 1 h. Antigen expression was observed using an enhanced chemiluminescence kit. The following primary antibodies were used: anti-P-gp (1:1,000), anti-CYP2C19 (1:1,000), anti-IL-1β (1:2,000), anti-NF-κB-p65 (1:50,000), anti-TLR4 (1:1,000), anti-MyD88 (1:1,000), anti-NF-κB (1:1,000), anti-GAPDH (1:10,000), anti-His-H3 (1:6,000), anti-TRAF6 (1:1,000) and anti-IL-1β-p17 (1:1,000).

### Statistical Analysis

Data were analyzed using GraphPad Prism version 8.0. One-way analysis of variance (ANOVA) and t-tests were used to compare differences among treatment means. Linear regression analysis was performed using the loss minimization (LM) function in R 3.5.3 (R Core Team, Vienna, Austria). Data are presented as means ± standard deviation (SD). All statistical tests were two-sided and differences were considered statistically significant at *p* < 0.05.

## Results

Demographic information is shown in Supplementary Table S2.

### The Metabolic Ability of Voriconazole Decreased During Inflammation

**FIGURE 1:**
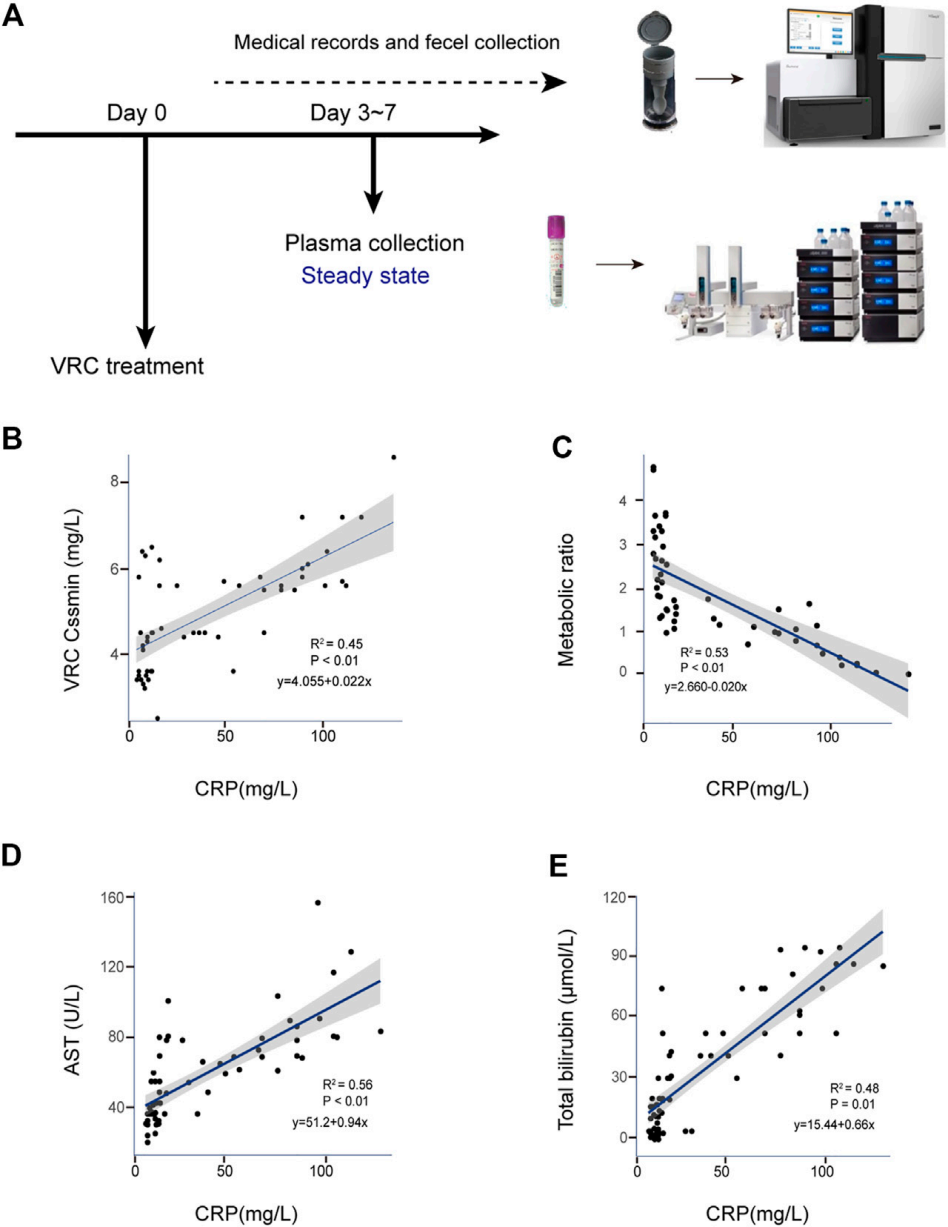
Correlation between CRP and adverse reactions in clinical voriconazole administration. **(A)** Schematic of sample collection process. **(B)** Scatterplot of VRC Cssmin *vs.* CRP concentration (mg/L). **(C)** Scatterplot of VRC metabolic ability *vs.* CRP concentration (mg/L). **(D,E)** Correlation between the CRP and liver function parameters possibly induced by voriconazole in peripheral blood. Each graph presents a trend line, regression equations, R-squared values (R^2^) and *p*-values of the regression model are shown on the figures (*n* = 65).

Sixty-five VRC trough concentrations were included in the analysis. There was a significant positive linear correlation between levels of the inflammatory marker CRP and VRC Cssmin (*R* ^2^ = 0.45; *p* < 0.001), Figure 1B. CRP levels were significantly positively correlated with AST (*R* ^2^ = 0.56; *p* < 0.001) (Figure 1D) and total bilirubin (*R* ^2^ = 0.48; *p* < 0.001) (Figure 1E).

To explore the ability of CRP to metabolize VRC in liver, we quantified nitrogen oxides metabolized by VRC, calculated the metabolic capacity, and established the correlation between CRP and metabolic ability. Figure 1C shows all calculated metabolic ratios and indicates that they decreased with increasing CRP concentration. CRP levels were significantly correlated with metabolic ratios (*R* ^2^ = 0.53; *p* < 0.001).

**FIGURE 2:**
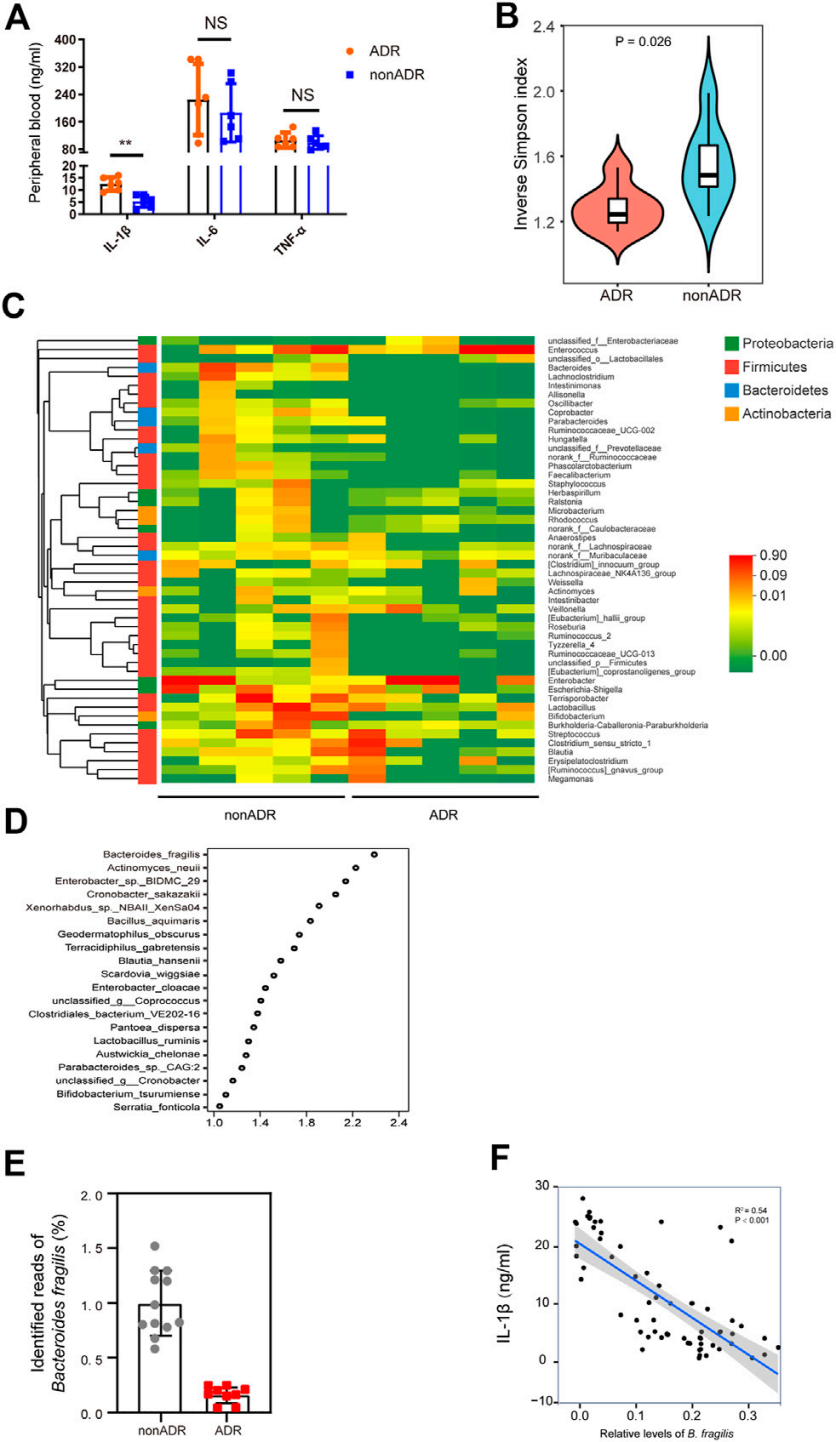
Profiling of the gut microbiota and pro-inflammatory cytokines in individuals with AML. **(A)** Pro-inflammatory cytokine concentrations were determined in peripheral blood (*n* = 6, respectively). **(B)** Alpha diversity, measured by the inverse Simpson index (*n* = 6, respectively). **(C)** Community heatmap analysis at genus level (*n* = 5, respectively). **(D)** VIP scores of PLS-DA. VIP scores ranked discriminating power of various taxa between nonADR and ADR groups. Taxon with VIP score of > 1 was considered important in discrimination. **(E)** Identified reads of *B. fragilis* in ADR and nonADR patients. **(F)** Scatterplots of IL-1β levels in periheral blood *vs.* relative levels of *B. fragilis*. *R* ^2^ and *p*-values of the regression model are shown on the figures.

Based on the correlation between inflammation and ADR detailed above, we also analyzed the correlations between key inflammatory cytokines and *B. fragilis* in the two groups. We found that ADR were associated with higher levels of IL-1β in peripheral blood (Figure 2A).

### *Bacteroides Fragilis* Abundance Was Higher in Patients Without Adverse Reactions

Alterations in patients’ microbial community structure and diversity may be associated with chemotherapy efficacy and toxicity (Jia et al., 2008). To determine whether gut microbial community diversity was altered in the AML individuals susceptible to ADRs, we evaluated **α**-diversity in the feces of nonADR and ADR patients using an inverse Simpson index, which is an overall diversity parameter (Figure 2B). Gut microbial abundance among groups was compared using a Wilcoxon rank sum test. At the phylum level, the abundance of *Bacteroides* was significantly lower in ADR patients than in nonADR patients (*p* = 0.025; Figure 2B).

Samples were randomly selected from each group and the relative differences in their genus-level *Bacteroides* abundance were analyzed. The heat map in Figure 2C shows that *Bacteroides* abundance was significantly higher in nonADR patients than in ADR patients. Variable importance in projection (VIP) scores for gut microbiota showed that *B. fragilis* contributed significantly to the group separation (Figure 2D). Identified reads of bacteria in the 16S rDNA data extracted from these fecal samples are presented in Figure 2E. Significant *B. fragilis* enrichment was found in the fecal samples obtained from AML patients presenting with no adverse reactions.

Based on the correlation between inflammation and ADRs outlined above, we also analyzed the correlations between key inflammatory cytokines and *B. fragilis* in the two groups. Of interest, IL-1β levels were negatively correlated with relative *B. fragilis* abundance (*R* ^2^ = 0.58; *p* < 0.001) (Figure 2F). This finding, accords with the results presented in Figure 2A.

### *Bacteroides Fragilis* Polysaccharide a Treatment Ameliorated Abnormal Hepatic Voriconazole Metabolism

**FIGURE 3:**
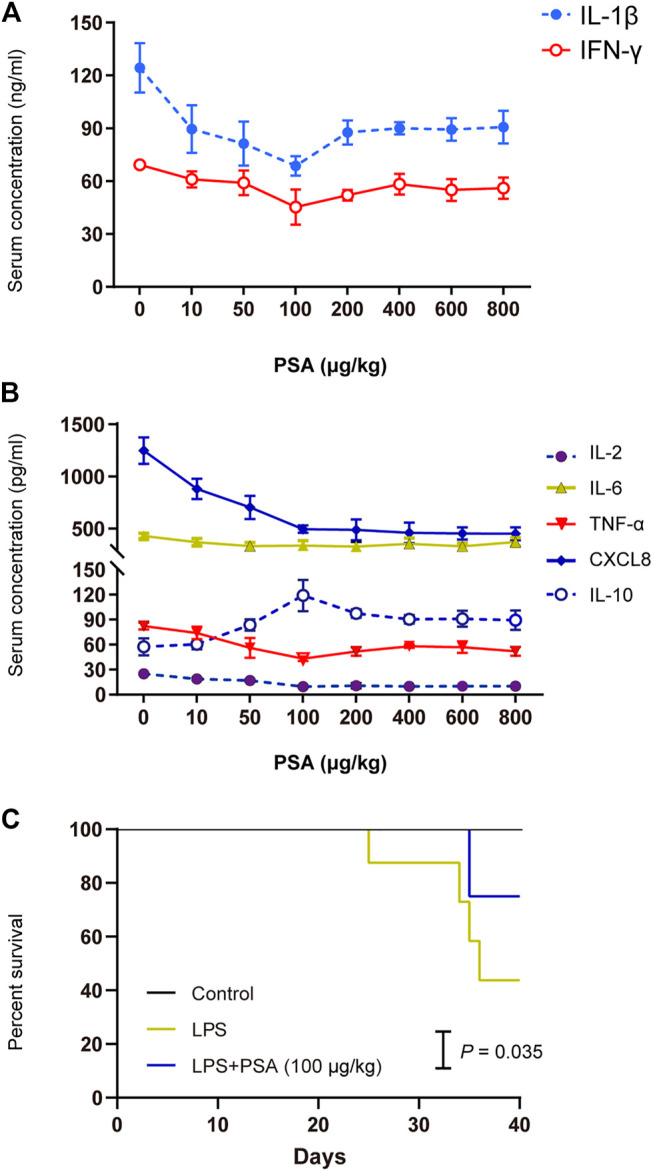
Concentration screening over the effect of PSA on animal models. **(A,B)** Screening assay was performed at eight PSA concentrations (0, 10, 50, 100, 200,400, 600 and 800 μg/kg) in animals (*n* = 5, respectively). Optimum PSA concentration selection based on the levels of proinflammatory cytokines. **(C)** Kaplan-Meier survival curve analysis with PSA (100 μg/kg).

We began by determining the appropriate dose of PSA for the animals (Figure 3). Based on the experimental analysis shown in Figures 3A,B, we set the dose for mice at 100 μg/kg, and that for rats at 50 μg/kg. Mouse survival analysis showed that PSA (100 μg/kg) significantly prolonged the survival times in the of LPS-induced model (Figure 3C).

**FIGURE 4:**
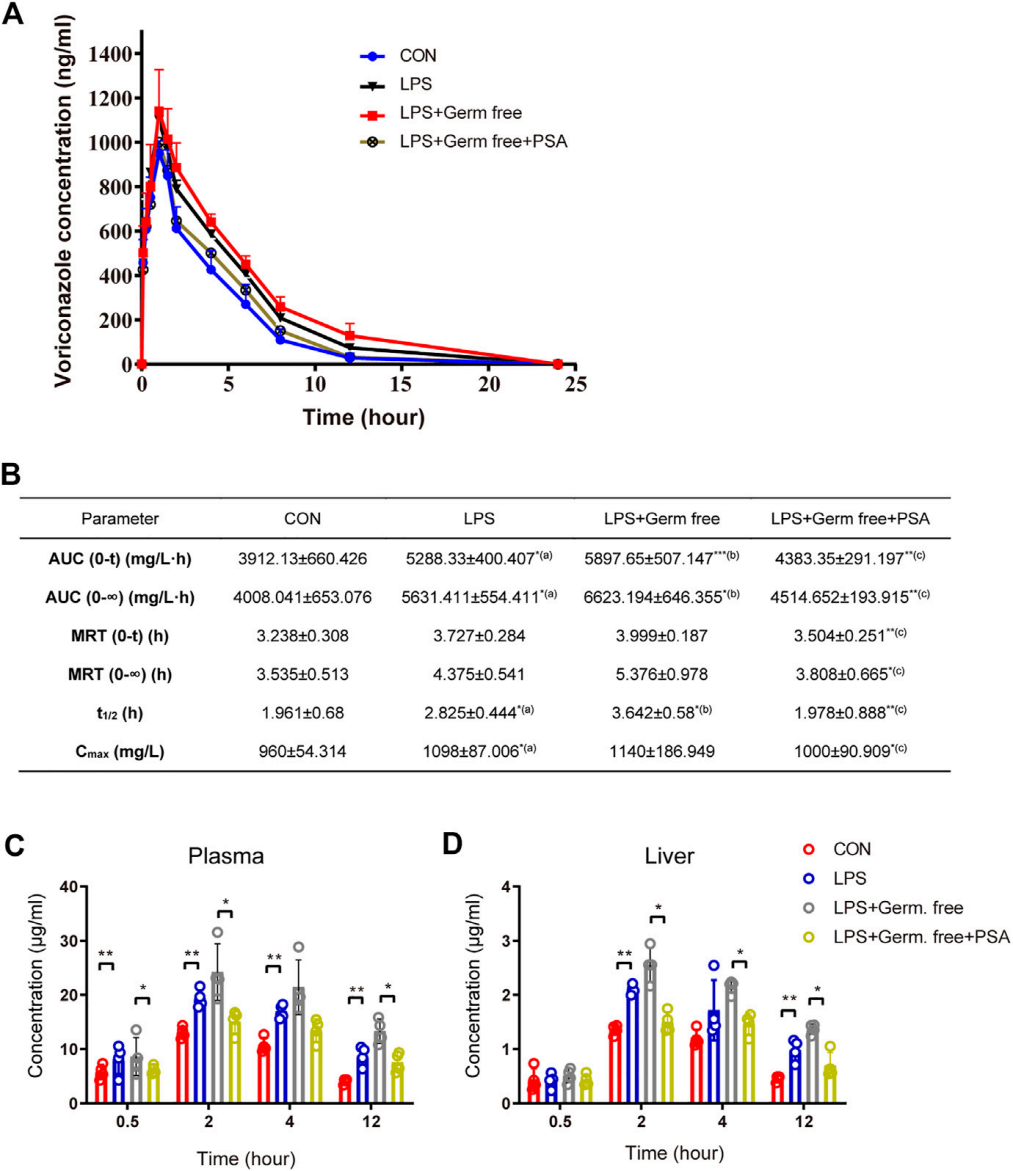
VRC metabolism by LPS with or without PSA treated rats and mice. **(A)** VRC plasma kinetics in individual rats (*n* = 5, respectively). **(B)** Pharmacokinetic (PK) parameters in rats. **(C)** VRC blood concentrations in individual mice (*n* = 5, respectively). **(D)** VRC liver tissue concentrations in individual mice. The PK parameters were calculated according to the noncompartmental PK calculated by DAS 2.0 software. Tmax, maximum time corresponding to Cmax; AUC (0-∞), area under the curve from 0 to ∞; MRT, Mean residence time; t1/2, half-life; Cmax, maximum concentration; Rats receiving a single dose of VRC, 10 mg/kg; Mice receiving a single dose of VRC, 10 mg/kg. (a) LPS *vs.* CON; (b) LPS + Germ free *vs.* LPS; (c) LPS + Germ free + PSA *vs.* LPS + Germ free. Unpaired *t* test, *p < 0.05, **p < 0.01, ***p < 0.001.

In the rat experiment, we verified that metabolism of VRC was abnormal in the inflammatory-model animals. The VRC metabolism drug-time curves for each group are shown in Figure 4A, which shows that VRC AUC and plasma disposal ability were significantly increased in the LPS-treated rats, as well as other pharmacokinetic (PK) parameters (Figure 4B), implying that inflammation increased the risk of VRC induced adverse reactions. PSA treatment significantly improved VRC AUC and other PK parameters.

In the mouse experiment, we monitored the dose-time relation of voriconazole in the liver and serum and again found that PSA alleviated the abnormal metabolism of voriconazole in the inflammatory-model animals (Figures 4C,D). PSA significantly reduced the peak concentration of voriconazole within 2 h. At 12 h, the concentrations of voriconazole in the liver and serum were significantly reduced (*p* < 0.05). These results imply that PSA was beneficial in reducing the residual amounts of voriconazole during metabolic clearance *in vivo*.

PSA administration also reversed VRC-induced hepatotoxicity in the inflammatory-model animals (Figures 4A–C). Levels of AST, ALT and TB were significantly lower in the PSA-treated group than in the LPS-treated group.

### Polysaccharide A Reversed Hepatic Inflammatory Lesions

PSA is the main functional molecule produced by *B. fragilis*. It has a zwitterionic structure consisting of repeating oligosaccharide units comprising sugars with free amino and carboxyl groups. It is vital to the biological activity of bacteria (Supplementary Figure S1). To determine the optimal cellular and molecular concentrations required for an immune response to PSA *in vivo*, the effects of purified PSA on IL-1β and IFN-γ levels in mice were assessed. At 0–100 μg/kg, PSA decreased IL-1β and IFN-γ levels. At > 100 μg/kg, however, PSA was no longer inhibitive (Figure 3A). We selected a concentration of 100 μg/kg PSA for the mouse gastric-perfusion experiment.

**FIGURE 5:**
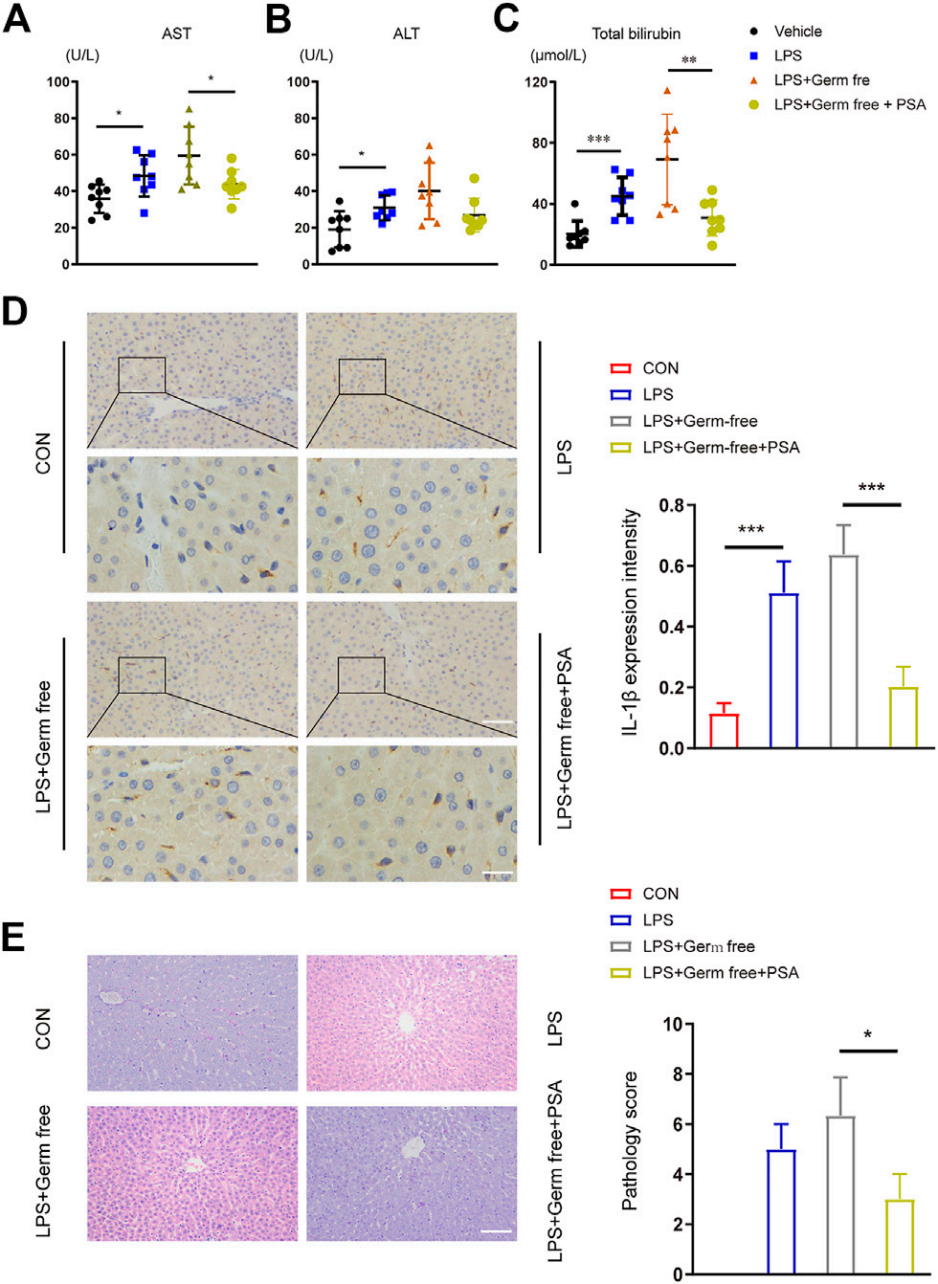
Hepatic function and histological analysis of the livers after intravenous administration of LPS with or without PSA in mice. **(A–C)** Plasma chemistry profile analyses of aspartate transaminase (AST) **(A)** alanine transaminase (ALT) **(B)** and total bilirubin **(C)** after VRC treated 5 days **(D)** Left: Immunohistochemical (IHC) detection of hepatic IL-1β (200 × upper and 400 × down), Right: IL-1β expression intensity in different treated groups were analyzed **(E)** Left: Photomicrographs of stained with H&E (200×). Right: Semiquantitative pathology scores of liver tissues. *p < 0.05, **p < 0.01, ***p < 0.001; Unpaired *t* test.

After continuous intragastric PSA administration, the livers of the mice were excised for pathological examination. Continuous intragastric PSA administration significantly improved the inflammatory response and reduced the edematous liver tissue degeneration induced by LPS (Figure 5E). We then performed IHC experiments on the liver tissue and found that the IL-1β secretion in the PSA treated group did not differ from that in the normal control (CON) group, but was lower than that in the group with inflammation induced by LPS (Figure 5D).

### Polysaccharide A Inhibits the Secretion of IL-1β Through the TLR4 Signaling Pathway

**FIGURE 6:**
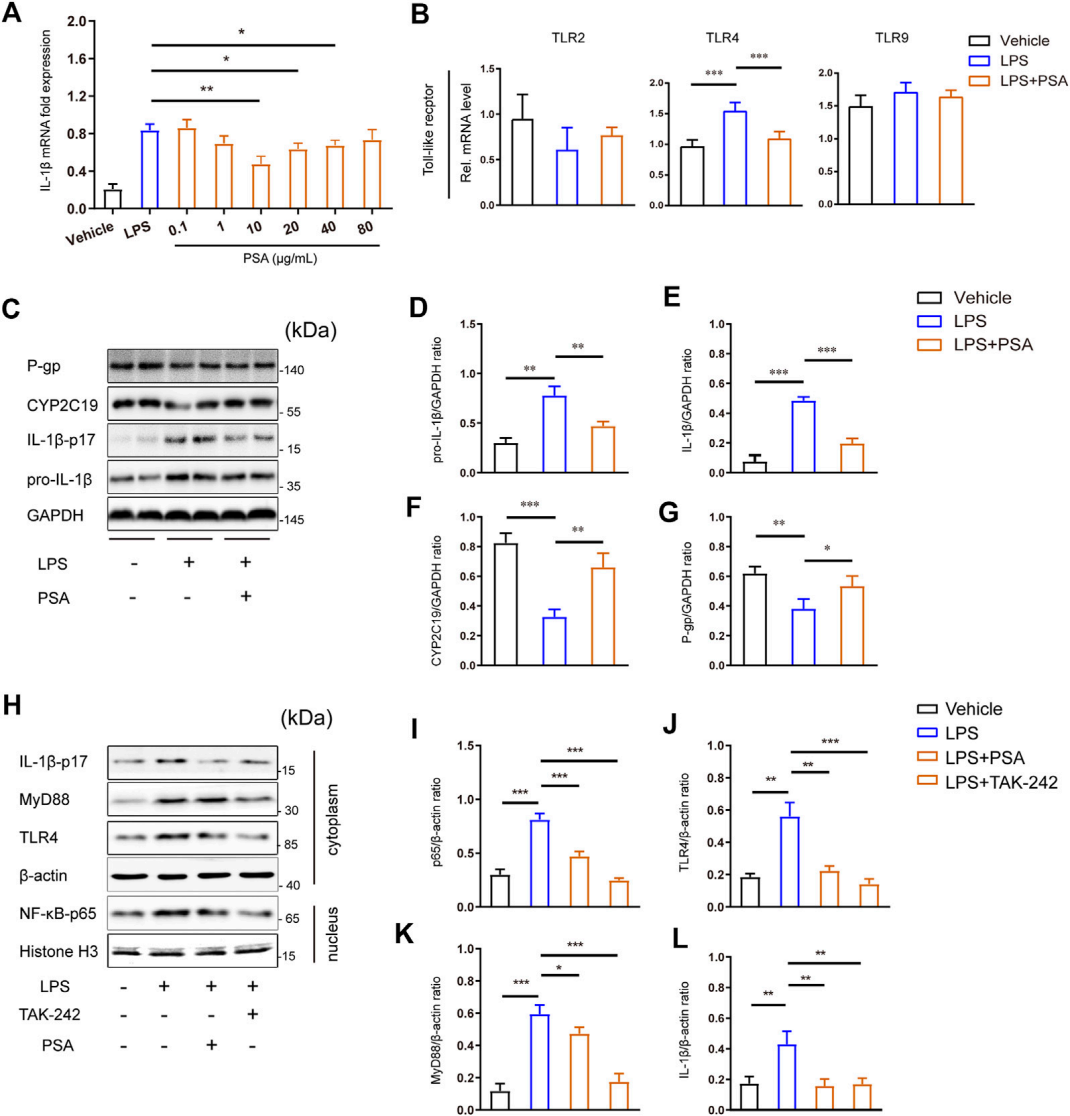
PSA inhibits the secretion of IL-1β may through TLR4 signal pathway. **(A)** Optimum PSA concentration selection based on the mRNA expression levels of IL-1β. **(B)** mRNA levels of key toll-like recepters in the liver (*n* = 6–12). **(C–G)** Immunblots **(C)** and densitometric analysis of pro-IL-1β **(D)**, IL-1β-p17 **(E)**, CYP2C19 **(F)** and P-gp **(G)** in liver tissue homogenates treated with the vehicle, LPS and LPS + PSA. **(G–K)**, Immunblots **(H)** of key proteins of TLR4 signal transduction and densitometric analysis of these proteins **(I–L)** in primary hepatocyte treated with the vehicle, LPS, LPS + PSA and LPS + TAK-242. Western blot results are representative of three independent experiments. *p < 0.05, **p < 0.01, ***p < 0.001, Unpaired *t* test.

To confirm the foregoing results *in vivo*, we used western blotting to measure hepatic inflammasome suppression and drug enzyme and transporter protein upregulation in response to PSA treatment. Here, we specifically analyzed IL-1β, voriconazole metabolic enzyme (CYP2C19) and transporter (P-gp). Following PSA administration, the mRNA expression levels of IL-1β decreased in a dose-dependent manner, and this was reversed at a PSA concentration of 10 μg/ml, the proteins were immunostained in primary hepatocytes (Figure 6C). As expected, LPS stimulation induced significant IL-1β-17 protein secretion (Figures 6C–E). CYP2C19 and P-gp expression were down-regulated after LPS treatment. Remarkably, the western blot analysis showed that PSA treatment significantly reduced IL-1β protein expression, whereas that of CYP2C19 and P-gp significantly increased (Figures 6F,G).

To elucidate the mechanisms involved, we analyzed toll-like receptors TLR gene expression in primary hepatocytes and found that PSA upregulated TLR4 significantly after LPS stimulation (Figure 6B). Inhibition of the hepatic inflammatory response by PSA may be associated with down-regulation of the TLR4 signaling pathway. We therefore used TAK-242 to disrupt TLR4 signaling in primary hepatocytes. When TLR4 signaling was blocked in primary hepatocytes stimulated by LPS, there was a significant (*p* < 0.001) decrease in MyD88 and IL-1β protein levels (Figures 6K,L); to a lesser extent, NF-κB levels were also reduced (Figure 6I). Of note, a similar observation was made in PSA-treated primary hepatocytes: TLR4, MyD88, IL-1β and NF-κB-p65 were significantly decreased (*p* < 0.05). This may indicate that PSA can play a similar role to that of TAK-242 targeting the same target.

### Polysaccharide A Up Regulates Expression of Hepatic CYP2C19 and P-gp by Inhibiting the TLR4/NF-κB Signaling Pathway

**FIGURE 7:**
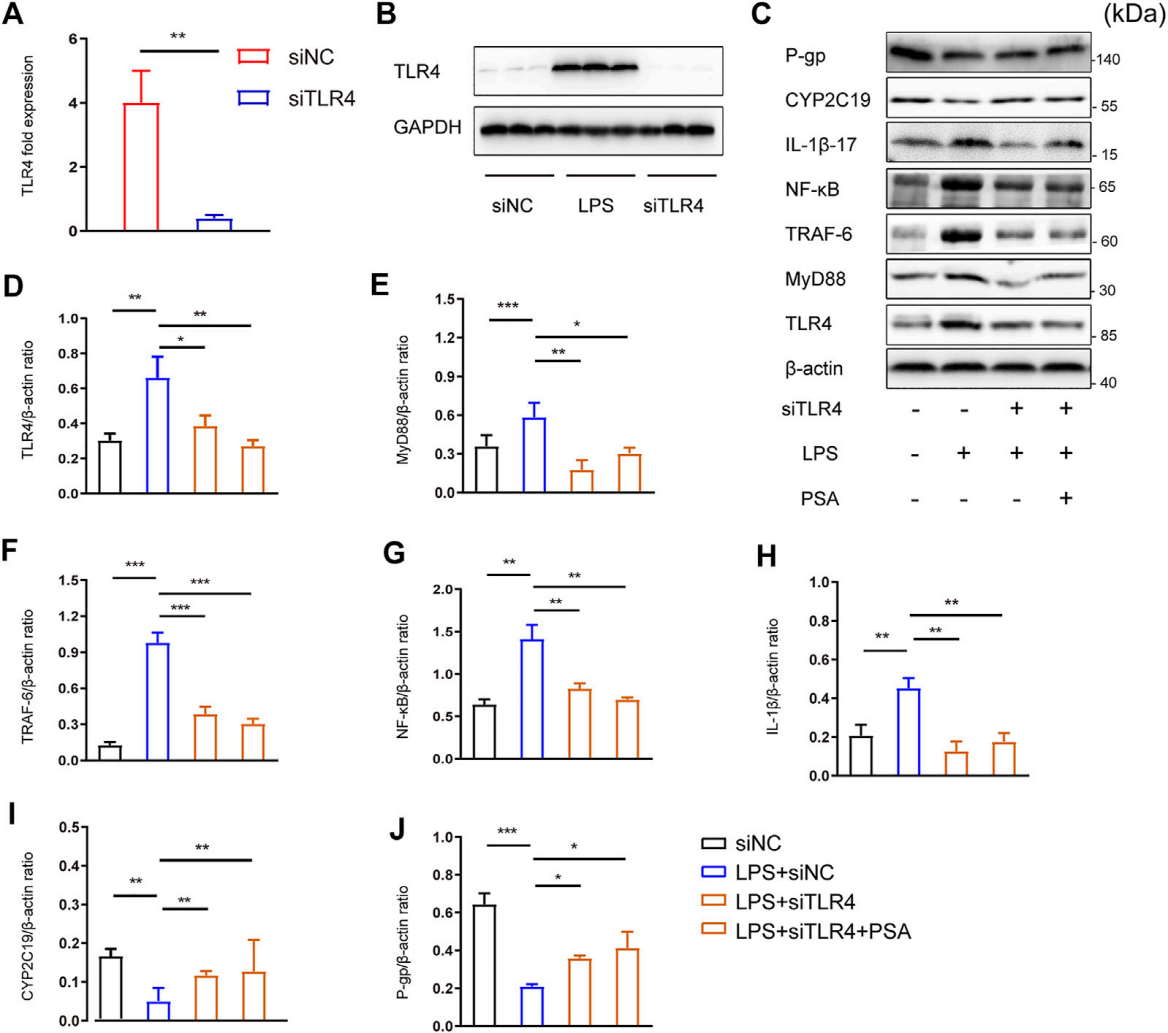
PSA up-regulates the expression of hepatic CYP2C19 and P-gp by inhibiting TLR4/NF-κB signal pathway. **(A,B)** RT-PCR and Western blot analyses were used to evaluate the efficiency of TLR4 knock-down by siRNA (**p < 0.01) **(C–H)** Immunblots **(C)** and densitometric analysis of TLR4 **(D)**, MyD88 **(E)**, TRAF6 **(F)**, NF-κB **(G)** and IL-1β **(H)** in primary hepatocytes treated with the vehicle, LPS, LPS + siTLR4 and LPS + PSA **(C,I–J)**, Immunblots **(C)** and densitometric analysis of CYP2C19 **(I)**, and P-gp **(J)** in primary hepatocytes. Western blot results represent three independent experiments. *p < 0.05, **p < 0.01, ***p < 0.001, *t*-test.

To investigate the potential role of PSA in the TLR4 signaling pathway, lentivirus encoding siRNA against TLR4 (siTLR4) was used to selectively reduce TLR4 gene expression in primary hepatocytes. Scramble siRNA was used as a control (siNC) (Figures 7A,B).

There were no significant differences in the expression of CYP2C19 or P-gp between the LPS + siTLR4 and LPS + siTLR4+PSA groups (Figures 7C,I,J). PSA did not further increase expression of these proteins when TLR4 was silenced. These results imply that PSA affects the NF-κB signaling pathway *via* TLR4.

Comparatively, PSA reduced the relative protein levels of the main molecules in the TLR4/NF-κB signaling pathway. Infecting cells with siTLR4 and exposing them to LPS significantly decreased expression of MyD88, TRAF6 and NF-κB, three key proteins downstream of TLR4 (Figures 7C,E–G). Similarly, under the same conditions, MyD88, TRAF6 and NF-κB expression did not significantly decrease in response to PSA alone. More importantly, PSA did not further increase IL-1β expression when TLR4 was silenced. These findings suggested that PSA may affect IL-1β expression *via* the TLR4/NF-κB signaling pathway, thus regulating the expression of metabolic enzymes.

## Discussion

**FIGURE 8:**
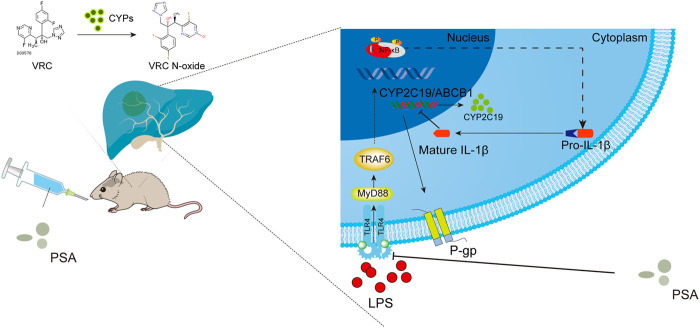
Schematic illustration of PSA regulatory mechanism of VRC metabolism. PSA inhibits the TLR4/NF-κB axis signal transduction pathway induced by LPS, suppressed the secretion of mature IL-1β in the cytoplasm, promotes the expression of liver drug metabolic enzyme (CYP2C19) and transporter (P-gp and its protein coding gene ABCB1), and finally improves the metabolic ability of voriconazole.

The aim of this study was to determine whether the immunomodulatory molecule, PSA, from intestinal *B. fragilis* mitigates individual differences in VRC metabolism among patients with inflammation by regulating hepatic metabolic enzyme activity. Analysis of correlations between CRP and abnormal VRC metabolism in clinical cases revealed that hepatotoxicity and weak VRC metabolic ability significantly increased with CRP levels. Metagenome sequence analysis revealed that high *B. fragilis* abundance was strongly related to the inhibition of pro-inflammatory cytokines such as IL-1β. We established a sterile inflammatory disease model to elucidate the mechanism by which *B. fragilis* natural product capsular (PSA) alleviates abnormal VRC metabolism in an inflammatory environment. Molecular studies showed that PSA downregulated the LPS-induced IL-1β expression by inhibiting the TLR4/NF-kB signaling pathway. The former would alter the enzymatic expression of P-450 isoform (CYP2C19), as well as regulate drug transporters such as hepatic P-glycoprotein, ultimately affecting VRC metabolism. Figure 8 shows the major modes of action identified in this study.

Gene polymorphism may reflect differences in susceptibility to drug treatment between individuals (Mangal et al., 2018; Zhuo et al., 2020). Here, there was still inter-individual variability in VRC concentrations and hepatotoxicity even among patients of the same metabolic type. In the past 2 decades, studies on the effects of treatment-related gastrointestinal changes, protein-binding changes, drug-drug interactions and even food intake on voriconazole metabolism have been reported (Purkins et al., 2003; Hope et al., 2013; Yuan et al., 2020). While it has been shown that these physiological characteristics affect individual differences in response to voriconazole, the influence of inflammatory responses (*eg.,* CRP, IL-6) in combination with these physiological characteristics on the course of disease has not been investigated. Furthermore, there has been no report on the effect of “3 + 7” therapy on voriconazole metabolism, or the effects of daunorubicin and cytarabine on voriconazole metabolism. On the other hand, one study found that intake of whole grains, fiber and healthy fats reduced systemic inflammation, and therefore represents a promising treatment for a deleterious inflammatory response (Bujtor et al., 2021). A high-quality diet may also alleviate the metabolic disorders associated with voriconazole. Therefore, patients with unconventional or unhealthy eating habits were excluded from this study. Although a prospective study reported that voriconazole metabolism is affected by severe inflammation (Veringa et al., 2017), the present study is the first to report clinical analysis samples from AML patients taking VRC. This has clarified the influences of CRP on individual differences observed in VRC concentrations, furthermore, we focused on a single disease (AML) and a specific treatment (“3 + 7”) to develop a microbiome-based therapy.

The liver promotes C-reactive protein production in hepatocytes by excreting circulating triglycerides and free fatty acids and triggering the release of cytokines which play key roles in inflammation (Zhong et al., 2020). The observed diminution in VRC metabolism during inflammation may be explained by the concomitant synthesis of hub targets of the active compounds, namely TNF-α, IL-1β and IL-6 which in turn, alter the expression of specific transcription factors such as NF-κB, leading to a vicious circle of inflammatory response (Zhou et al., 2020). Our results support the concept that disruption of metabolic capacity is accompanied by an increased in liver damage due to inflammation, CRP levels increased with the VRC exposure level. We found that patients prone to ADRs had significantly lower *B. fragilis* abundance than those who were not susceptible to them. Whole-metagenome sequence analysis also showed that *B. fragilis* abundance was negatively correlated with the levels of pro-inflammatory cytokines such as IL-1β. Hence, *B. fragilis* has a beneficial immunomodulatory effect on hosts undergoing VRC therapy. The results of this study are consistent with the concept that *B. fragilis* strongly reverses host susceptibility to inflammatory diseases (Sun et al., 2019).

Clinical findings need to be verified by basic experiments. We developed a sterile inflammatory disease model to elucidate the regulatory events responsible for the regulation of DMET gene expression by PSA. A recent study showed that oral PSA induced intestinal regulatory cells and prevented pro-inflammatory activity (Mazmanian et al., 2008; Ramakrishna et al., 2019). It was also shown to alleviate respiratory tract inflammations, encephalitis, and colitis (Sun et al., 2019). Here, we explored whether PSA would alleviate VRC hepatotoxicity in sterile inflammatory model. As expected, *B. fragilis* natural product ameliorated hepatic lesions and mitigated VRC hepatotoxicity. In the experiment using primary hepatocytes, we found that PSA significantly down-regulated the mRNA expression of TLR4 in toll-like receptor induced by LPS stimulation. Moreover, as shown in Figure 6C, with the decrease in IL-1β secretory protein in the PSA treated group, expression of CYP2C19 and P-gp increased. We speculate that *B. fragilis* PSA improves abnormal hepatic VRC metabolism induced by TLR4, which may trigger inflammatory cytokine production.

Notably, IL-1β is a central mediator of innate immunity and inflammation. TLRs induce activation of the NF-κB/MAP kinase (MAPK) signaling pathway, which leads to the transcription of genes encoding inflammatory cytokines (IL-1β and TNF-α), chemokines, cyclooxygenase-2, and inducible NO synthase (Stanke-Labesque et al., 2020). Accumulating evidence indicates that production of IL-1β is closely correlated with TLR4 (Jiménez-Dalmaroni et al., 2016; Phongsisay, 2016). IL-1β is a pleiotropic cytokine with multiple roles in various physiological and pathological conditions (Jesus and Goldbach-Mansky, 2014). We propose that to intervene in LPS-induced abnormal hepatic VRC metabolism, it is desirable to target IL-1β. Administered PSA suppresses TLR-mediated NF-κB activation in the nucleus, by extension, inhibiting IL-1β secretion. Figure 6H shows that the expression of IL-1β decreased significantly when the TLR4 signaling pathway was blocked. Of note, compared with the TLR4 signal inhibitor TAK-242, PSA had the same effect on IL-1β inhibition. This further simplies that IL-1β is a downstream inflammatory cytokine in the TLR4/NF-κB signaling pathway.

Moreover, we examined the effect of TLR4 silencing on LPS-induced metabolic inhibition and the prospective molecular mechanisms. Our *in vitro* results indicate that TLR4 deficiency abrogated the increases in LPS-induced TLR4 and TRAF6 expression. Subsequently, TLR4 deficiency attenuated LPS-mediated changes in the proinflammatory cytokine IL-1β. Finally, TLR4 deficiency increased the expression of metabolic enzyme and transporter genes in primary hepatocytes. Similarly, in the siTLR4+PSA group, cellular levels of these proteins were the same as those in the siTLR4 group. These data imply that the inhibitory effect of PSA on inflammation is similar to that of TLR4/NF-κB signal transduction, ultimately inhibiting the secretion of the downstream inflammatory factor IL-1β and promoting the expression of hepatic drug metabolic enzymes.

Nevertheless, the present study had several limitations. First, as PSA is an amphoteric macromolecular compound, it must have some impact on intestinal immune regulation. It has been reported that binding to TLR2^+^ pDC and macrophages, causes tissue-specific regulatory T cells to secrete IL-10 after orally administered PSA (Shen et al., 2012; Dasgupta et al., 2014). Therefore, other correlations between intestinal and hepatic cellular immunity need to be elucidated. Second, to the best of our knowledge, this study is the first to verify the therapeutic action of PSA on hepatocytes’ inflammatory response *in vivo*. Hence, the efficacy of PSA should also be validated in other diseases models such as in animal models of AML. Further studies are also warranted to determine whether gut bacteria such as *B. fragilis* that are associated with abnormal hepatic metabolism play roles in immune dysfunction and liver inflammation in the AML pathogenesis.

The present study demonstrated that PSA up-regulates the expression of hepatocellular CYP2C19 and P-gp *via* TLR4-mediated NF-κB transcription and IL-1β expression. Our findings imply that, monitoring of the abundance of *B. fragilis* in feces during voriconazole treatment is recommended during and after severe inflammation, to reduce the risk of adverse reactions to voriconazole.

## Data Availability

The datasets generated for this study are available on request to the corresponding authors. The sequencing data in our study has been uploaded in the BioProject: PRJNA707356.

## References

[B1] Adolph T. E. Grander C. Moschen A. R. Tilg H. (2018). Liver-microbiome axis in health and disease. Trends Immunol. 39, 712–723. 10.1016/j.it.2018.05.002 29843959

[B2] Bujtor M. Turner A. Torres S. Esteban-Gonzalo L. Pariante C. Borsini A. (2021). Associations of dietary intake on biological markers of inflammation in children and adolescents: a systematic review. Nutrients 13, 356–384. 10.3390/nu13020356 33503979 PMC7911843

[B3] Dasgupta S. Erturk-Hasdemir D. Ochoa-Reparaz J. Reinecker H.-C. Kasper D. L. (2014). Plasmacytoid dendritic cells mediate anti-inflammatory responses to a gut commensal molecule via both innate and adaptive mechanisms. Cell Host Microbe. 15, 413–423. 10.1016/j.chom.2014.03.006 24721570 PMC4020153

[B4] Gong S. Yan Z. Liu Z. Niu M. Fang H. Li N. (2019). Intestinal microbiota mediates the susceptibility to polymicrobial sepsis‐induced liver injury by granisetron generation in mice. Hepatology 69, 1751–1767. 10.1002/hep.30361 30506577

[B5] Hicks J. K. Quilitz R. E. Komrokji R. S. Kubal T. E. Lancet J. E. Pasikhova Y. (2020). Prospective CYP2C19 ‐guided voriconazole prophylaxis in patients with neutropenic acute myeloid leukemia reduces the incidence of subtherapeutic antifungal plasma concentrations. Clin. Pharmacol. Ther. 107, 563–570. 10.1002/cpt.1641 31549389 PMC7018540

[B6] Hope W. W. Vanguilder M. Donnelly J. P. Blijlevens N. M. A. Brüggemann R. J. M. Jelliffe R. W. (2013). Software for dosage individualization of voriconazole for immunocompromised patients. Antimicrob. Agents Chemother. 57, 1888–1894. 10.1128/aac.02025-12 23380734 PMC3623322

[B7] Jesus A. A. Goldbach-Mansky R. (2014). IL-1 blockade in autoinflammatory syndromes. Annu. Rev. Med. 65, 223–244. 10.1146/annurev-med-061512-150641 24422572 PMC4178953

[B8] Jia W. Li H. Zhao L. Nicholson J. K. (2008). Gut microbiota: a potential new territory for drug targeting. Nat. Rev. Drug Discov. 7, 123–129. 10.1038/nrd2505 18239669

[B9] Jiménez-Dalmaroni M. J. Gerswhin M. E. Adamopoulos I. E. (2016). The critical role of toll-like receptors - from microbial recognition to autoimmunity: a comprehensive review. Autoimmun. Rev. 15, 1–8. 10.1016/j.autrev.2015.08.009 26299984 PMC4679489

[B10] Kawai T. Akira S. (2010). The role of pattern-recognition receptors in innate immunity: update on Toll-like receptors. Nat. Immunol. 11, 373–384. 10.1038/ni.1863 20404851

[B11] Keller R. Klein M. Thomas M. Dräger A. Metzger U. Templin M. (2016). Coordinating role of RXRα in downregulating hepatic detoxification during inflammation revealed by fuzzy-logic modeling. PLoS Comput. Biol. 12, e1004431. 10.1371/journal.pcbi.1004431 26727233 PMC4699813

[B12] Lee Y. K. Mazmanian S. K. (2010). Has the microbiota played a critical role in the evolution of the adaptive immune system?. Science 330, 1768–1773. 10.1126/science.1195568 21205662 PMC3159383

[B13] Mangal N. Hamadeh I. S. Arwood M. J. Cavallari L. H. Samant T. S. Klinker K. P. (2018). Optimization of voriconazole therapy for the treatment of invasive fungal infections in adults. Clin. Pharmacol. Ther. 104, 957–965. 10.1002/cpt.1012 29315506 PMC6037619

[B14] Mazmanian S. K. Round J. L. Kasper D. L. (2008). A microbial symbiosis factor prevents intestinal inflammatory disease. Nature 453, 620–625. 10.1038/nature07008 18509436

[B15] Morgan E. (2009). Impact of infectious and inflammatory disease on cytochrome P450-mediated drug metabolism and pharmacokinetics. Clin. Pharmacol. Ther. 85, 434–438. 10.1038/clpt.2008.302 19212314 PMC3139248

[B16] Pascual A. Csajka C. Buclin T. Bolay S. Bille J. Calandra T. (2012). Challenging recommended oral and intravenous voriconazole doses for improved efficacy and safety: population pharmacokinetics-based analysis of adult patients with invasive fungal infections. Clin. Infect. Dis. 55, 381–390. 10.1093/cid/cis437 22610925

[B17] Phongsisay V. (2016). The immunobiology of Campylobacter jejuni: innate immunity and autoimmune diseases. Immunobiology 221, 535–543. 10.1016/j.imbio.2015.12.005 26709064

[B18] Purkins L. Wood N. Kleinermans D. Greenhalgh K. Nichols D. (2003). Effect of food on the pharmacokinetics of multiple-dose oral voriconazole. Br. J. Clin. Pharmacol. 56, 17–23. 10.1046/j.1365-2125.2003.01994.x 14616409 PMC1884315

[B19] Ramakrishna C. Kujawski M. Chu H. Li L. Mazmanian S. Cantin E. (2019). Bacteroides fragilis polysaccharide A induces IL-10 secreting B and T cells that prevent viral encephalitis. Nat. Commun. 10, 2153. 10.1038/s41467-019-09884-6 31089128 PMC6517419

[B20] Shen Y. Torchia M. L. G. Lawson G. W. Karp C. L. Ashwell J. D. Mazmanian S. K. (2012). Outer membrane vesicles of a human commensal mediate immune regulation and disease protection. Cell Host Microbe. 12, 509–520. 10.1016/j.chom.2012.08.004 22999859 PMC3895402

[B21] Sivan A. Corrales L. Hubert N. Williams J. B. Aquino-Michaels K. Earley Z. M. (2015). Commensal Bifidobacterium promotes antitumor immunity and facilitates anti-PD-L1 efficacy. Science 350, 1084–1089. 10.1126/science.aac4255 26541606 PMC4873287

[B22] Stanke-Labesque F. Gautier-Veyret E. Chhun S. Guilhaumou R. (2020). Inflammation is a major regulator of drug metabolizing enzymes and transporters: consequences for the personalization of drug treatment. Pharmacol. Ther. 215, 107627. 10.1016/j.pharmthera.2020.107627 32659304 PMC7351663

[B23] Sun F. Zhang Q. Zhao J. Zhang H. Zhai Q. Chen W. (2019). A potential species of next-generation probiotics? The dark and light sides of Bacteroides fragilis in health. Food Res. Int. 126, 108590. 10.1016/j.foodres.2019.108590 31732047

[B24] Tallman M. S. Wang E. S. Altman J. K. Appelbaum F. R. Bhatt V. R. Bixby D. (2019). Acute myeloid leukemia, version 3.2019, NCCN clinical practice guidelines in oncology. J. Natl. Compr. Canc. Ne. 17, 721–749. 10.6004/jnccn.2019.0028 31200351

[B25] Taur Y. Xavier J. B. Lipuma L. Ubeda C. Goldberg J. Gobourne A. (2012). Intestinal domination and the risk of bacteremia in patients undergoing allogeneic hematopoietic stem cell transplantation. Clin. Infect. Dis. 55, 905–914. 10.1093/cid/cis580 22718773 PMC3657523

[B26] Taylor M. Flannigan K. Rahim H. Mohamud A. Lewis I. Hirota S. (2019). Vancomycin relieves mycophenolate mofetil-induced gastrointestinal toxicity by eliminating gut bacterial β-glucuronidase activity. Sci. Adv. 5, eaax2358. 10.1126/sciadv.aax2358 31457102 PMC6685722

[B27] Veringa A. Ter Avest M. Span L. F. R. van den Heuvel E. R. Touw D. J. Zijlstra J. G. (2017). Voriconazole metabolism is influenced by severe inflammation: a prospective study. J. Antimicrob. Chemother. 72, 261–267. 10.1093/jac/dkw349 27601292

[B28] Wang X. Zhao J. Wen T. Liao X. Luo B. (2020). Predictive value of FMO3 variants on plasma disposition and adverse reactions of oral voriconazole in febrile neutropenia. Pharmacology 106 (3-4), 202–210. 10.1159/000510327 32998136

[B29] Yuan Z. Qiao C. Yang Z. Yu L. Sun L. Qian Y. (2020). The impact of plasma protein binding characteristics and unbound concentration of voriconazole on its adverse drug reactions. Front. Pharmacol. 11, 505–515. 10.3389/fphar.2020.00505 32390847 PMC7194128

[B30] Zhong W. Gummesson A. Tebani A. Karlsson M. Hong M. Schwenk J. (2020). Whole-genome sequence association analysis of blood proteins in a longitudinal wellness cohort. Genome Med. 12, 53–68. 10.1186/s13073-020-00755-0 32576278 PMC7310558

[B31] Zhou S. Ai Z. Li W. You P. Wu C. Li L. (2020). Deciphering the pharmacological mechanisms of taohe-chengqi decoction extract against renal fibrosis through integrating Network pharmacology and experimental validation in vitro and in vivo. Front. Pharmacol. 11, 425–442. 10.3389/fphar.2020.00425] 32372953 PMC7176980

[B32] Zhuo Z. Zhou C. Fang Y. Zhu J. Lu H. Zhou H. (2020). Correlation between the genetic variants of base excision repair (BER) pathway genes and neuroblastoma susceptibility in eastern Chinese children. Cancer Commun. 40, 641–646. 10.1002/cac2.12088 PMC7668499 32780923

[B33] Zimmermann M. Zimmermann-Kogadeeva M. Wegmann R. Goodman A. (2019). Separating host and microbiome contributions to drug pharmacokinetics and toxicity. Science 363, 1–16. 10.1126/science.aat9931 PMC6533120 30733391

