# Supplementary material for: Bacteroides Fragilis Polysaccharide A Ameliorates Abnormal Voriconazole Metabolism Accompanied With the Inhibition of TLR4/NF-κB Pathway
Source: Front Pharmacol. 2021 Apr 15;12:663325. doi: 10.3389/fphar.2021.663325 (PMC8115215; doi:10.3389/fphar.2021.663325)
Supplement: Supplementary file 1 [file datasheet1.docx]

**Supplementary Figures**


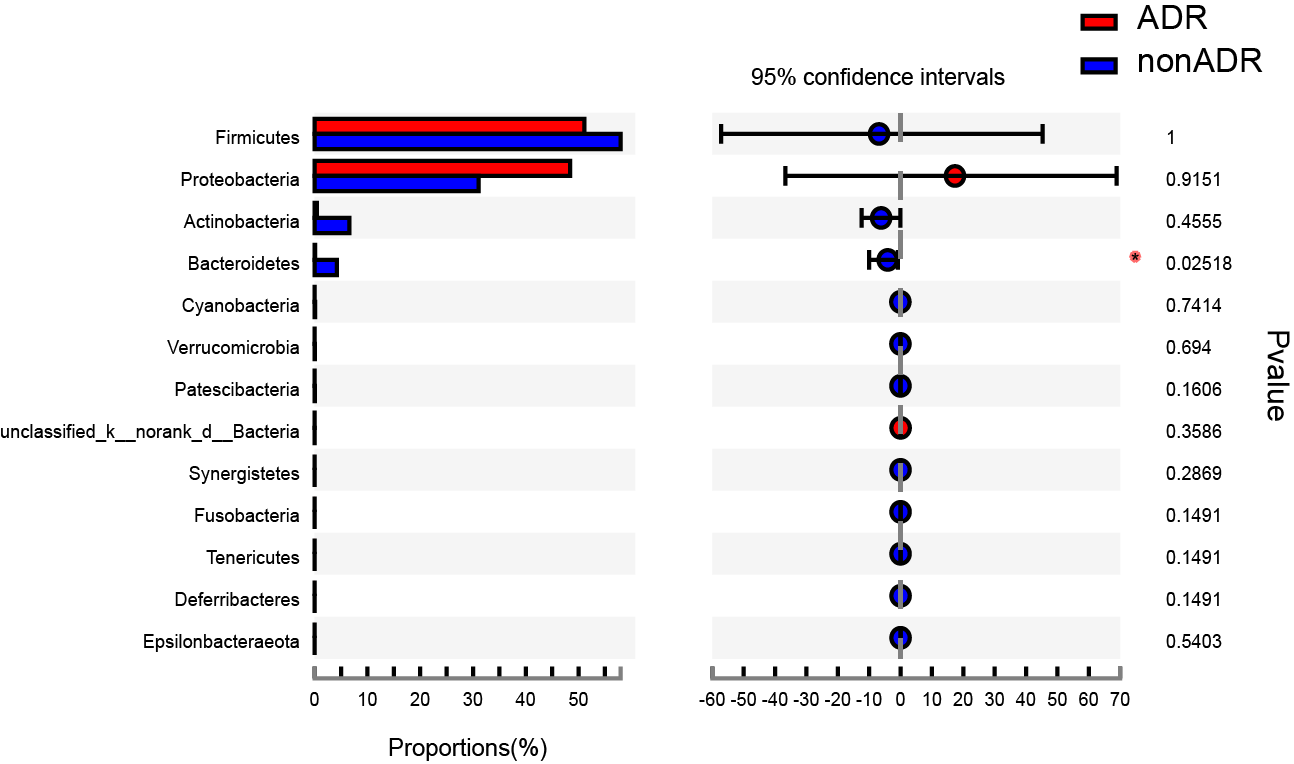


**Figure S1** Analysis of Wilcoxon rank-sum test bar plot of intestinal bacterial at phylum level of the corhorts. **P* < 0.05.


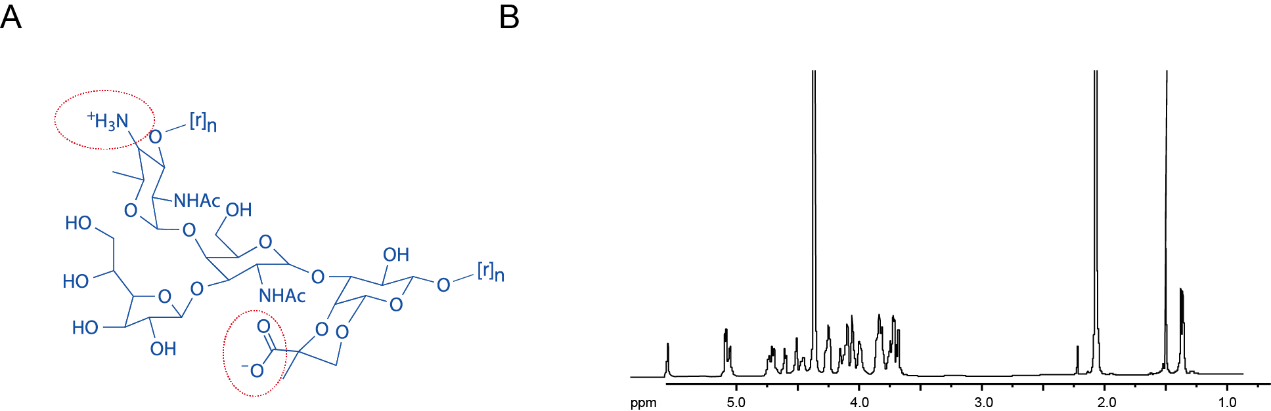


**Figure S2** Structure of polysaccharide A. Structure of polysaccharide A (PSA), the area shown in the circle is characterized by the zwitterionic structure of polysaccharides. It consists of a tetrasaccharide with free amino and carboxyl groups that confer zwitterionic behavior to this polymer. [r]n indicates where PSA subunit repeats branch off. B, ^1^H NMR spectra of PSA.
